# Intraspecific trait variation modulates the temperature effect on elemental quotas and stoichiometry in marine *Synechococcus*

**DOI:** 10.1371/journal.pone.0292337

**Published:** 2024-03-18

**Authors:** Renne Harcourt, Nathan S. Garcia, Adam C. Martiny

**Affiliations:** 1 Department of Ecology and Evolutionary Biology, University of California, Irvine, California, United States of America; 2 Department of Earth System Science, University of California, Irvine, California, United States of America; INRA/Sorbonne University, FRANCE

## Abstract

Diverse phytoplankton modulate the coupling between the ocean carbon and nutrient cycles through life-history traits such as cell size, elemental quotas, and ratios. Biodiversity is mostly considered at broad functional levels, but major phytoplankton lineages are themselves highly diverse. As an example, *Synechococcus* is found in nearly all ocean regions, and we demonstrate contains extensive intraspecific variation. Here, we grew four closely related *Synechococcus* isolates in serially transferred cultures across a range of temperatures (16–25°C) to quantify for the relative role of intraspecific trait variation vs. environmental change. We report differences in cell size (p<0.01) as a function of strain and clade (p<0.01). The carbon (*Q*_*C*_), nitrogen (*Q*_*N*_), and phosphorus (*Q*_*P*_) cell quotas all increased with cell size. Furthermore, cell size has an inverse relationship to growth rate. Within our experimental design, temperature alone had a weak physiological effect on cell quota and elemental ratios. Instead, we find systemic intraspecific variance of C:N:P, with cell size and N:P having an inverse relationship. Our results suggest a key role for intraspecific life history traits in determining elemental quotas and stoichiometry. Thus, the extensive biodiversity harbored within many lineages may modulate the impact of environmental change on ocean biogeochemical cycles.

## Introduction

Phytoplankton link the ocean carbon, nitrogen, and phosphorus cycles through the biomass ratios of these elements [1–4]. The Redfield ratio describes the oceanic carbon:nitrogen:phosphorus (C:N:P) stoichiometry and is used in models to assess export and productivity [5,6]. However, elemental ratios are variable in surface ecosystem [7,8], and ratios in the interior ocean may change over long-time scales, thereby influencing the ability of the oceans to sequester carbon relative to other nutrient elements [9]. Small cyanobacteria are currently estimated to account for approximately 25% of marine net primary production [10]. Therefore, a clear understanding of how key traits of marine cyanobacteria interact with environmental changes is needed to reduce uncertainties in ocean models of biogeochemistry and net primary production.

A number of factors are known to influence stoichiometry and elemental quotas. Latitudinal gradients in nutrient concentrations, temperature and community composition are dominant predictors of stoichiometry. However, their respective influences are difficult to decipher and accurately model as they strongly covary in the surface ocean. The nutrient hypothesis predicts that storage of phosphate (P) in polyphosphates [11] and nitrogen (N) storage in phycobiliproteins [12–15] are depleted under nutrient limitation, but elemental ratios approach an optimum under fast-growth when abundances and activities of components like P-rich ribosomes are high [2,16,17]. Temperature may directly influence the abundance of P-rich ribosomes in cells through a compensatory mechanism for reduced transcriptional activity under low temperature [18]. Future change is projected to cause an increase in sea surface temperatures globally [19]. It is important to assess physiological responses to temperature in a warning world.

Cellular elemental stoichiometry is also known to vary between major phylogenetic groups [20]. Cell size is a master trait that has significant implications for ecology and biogeochemistry, as a number of subordinal traits depend upon this key trait [21]. The dominance of small microbes in the open ocean facilitates rapid cycling of carbon by the microbial loop [22]. Sinking velocity increases with cell size, and aggregates of cells may form larger particles, which may more rapidly sink and lead to greater carbon sequestration [23]. The metabolic rate has been reported to scale inversely with cell size, with the degree of variance dependent on phylogeny [23–27]. Nutrient diffusion and uptake are enhanced in smaller cells due to their greater surface area to volume ratio [23,28]. Thus, cell size may influence elemental stoichiometry through these subordinal traits. In the open ocean, cell size varies inversely with population abundance [29], thereby exerting strong influences on ecology and biogeochemical models that rely on abundance [6,10,30–32]. If temperature deviates from the thermal optimum (T_opt_), variation in cell size may occur [23]. Key factors such as temperature and nutrient availability are considered major factors in determining certain biogeochemical estimates, such as C:N:P and net primary production, but it is also important to consider cell size [10].

At the genus level, *Synechococcus* is one of the most productive lineages in the global ocean and plays an important role at the base of food webs along broad nutrient and thermal gradients [10]. Within *Synechococcus*, multiple clades have been cited as being differently thermally adapted, with some clades more dominant in cold, nutrient-rich water relative to others [33,34]. Variable thermal optima for growth may be key in contributing to differences in biogeographical dominance of clades [35] but unknown traits may influence its biogeography. Variability in cell size among lineages of *Synechococcus* is known to exist [2,35] but has not been thoroughly examined despite the roles that cell size may play in determining cellular growth rate (*μ*), elemental quotas, and stoichiometry.

Here, we ask the following questions: 1) How does cell size vary between lineages of *Synechococcus*? 2) Are elemental quotas and ratios systematically different between strains of *Synechococcus*? 3) How does temperature influence growth rate, elemental quotas, and ratios of *Synechococcus*? We selected temperature as a factor due to assess the response of phytoplankton to well-documented current and projected anthropogenic increases in sea surface temperature [36,37]. We hypothesized cell size, stoichiometry, and elemental quotas would all be closely and strongly linked to *Synechococcus* strain identity. We also hypothesized strong thermal effects on stoichiometry, elemental quotas, and growth rate, as per the translation-compensation hypothesis, with a greater amount of phosphorus found in cells with elevated growth rates or under lower experimental temperatures. To test these hypotheses, we examined four *Synechococcus* strains, representing a gradient in cell size, from two cold-adapted clades isolated from three locations across the world: CC9902, BL107 (clade IV), CC9311, and ROS8604 (clade I) [38–41]. We selected four strains from two-cold adapted clades to investigate potential differences in size, cell quotas, growth rate, and stoichiometry that evolutionary thermal adaptations alone cannot account for. We present new findings about the potential link between cell size and phylogeny of *Synechococcus*, raising new questions about the ecology and biogeochemistry of picocyanobacteria.

## Materials and methods

### Strain information

#### Culture conditions

**Fig 1:**
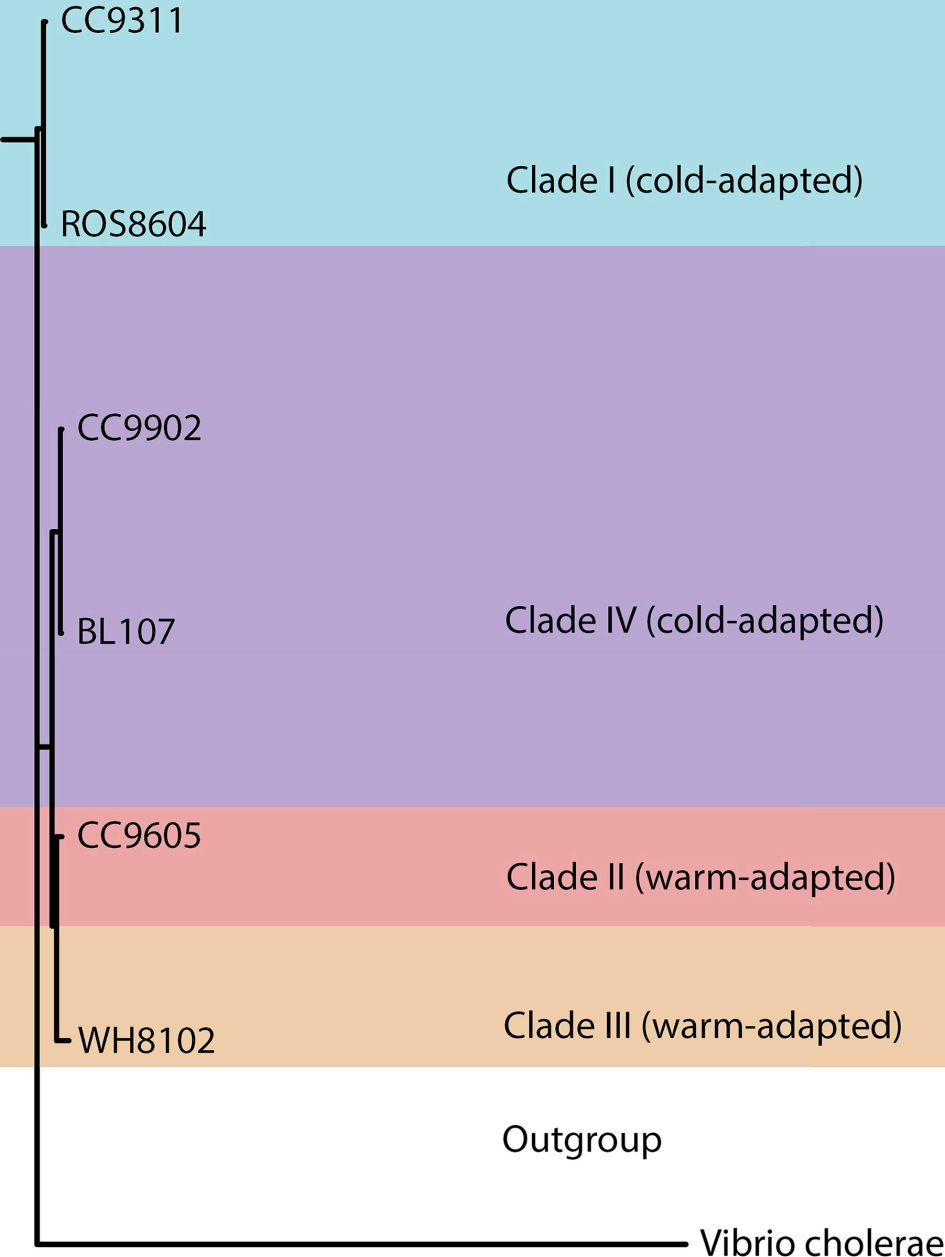
Phylogeny of examined *Synechococcus* strains. Color represents clades and the outgroup. *Vibrio cholerae* serves as the outgroup for constructed *Synechococcus* phylogeny. CC9605 and WH8102 represent clades II and III, respectively.

We incubated serially transferred cultures of *Synechococcus* (CC9311, BL107, ROS8604, CC9902, representing two clades) (Fig 1, Table 1) in triplicate 1 L flasks at 16°C, 18°C, 20°C, 22°C, 25°C, and 27°C. We supplied ambient light (60 μmol quanta m^-2^ s^-1^) using white fluorescent lamps on a 12:12 light-dark cycle. Culture media (modified artificial sea water) was prepared as described in Garcia et al. (2016) [2]. To ascertain that we did not impose growth rates upon cultures and the observed growth rates are a product of strain and environmental conditions, we used nutrient replete media and sampled prior to cultures encountering nutrient limitation; we supplied nitrate (NO_3_^-^) and phosphate (PO_4_^3−^) in concentrations of 125 μM and 10 μM, respectively. Cells utilized a mean of 17% of the supplied nitrogen, and 22% of the supplied phosphorus, indicating cultures did not reach nutrient limitation. We transferred media and diluted cultures using an open flame in a hood in order to avoid contamination. We diluted weekly by approximately an order of magnitude to avoid nutrient limitation and maintain a stable growth rate. While other questions, such as the role nutrient limitation and temperature may serve in determining physiological responses in these strains of *Synechococcus*, may be posed, chemostats (a continuous culture method) are likely better suited to answer such questions rather than our serial transfer methodology [2].

**Table 1:** *Synechococcus* strain information. The strains used during experimentation are presented here, with additional information such as clade, location of isolation, isolation depth, previously reported temperature optimum, our experimentally observed thermal optimum, previously reported thermal ranges, our experimentally observed range in cell size, our experimentally observed mean cell size, growth in oligotrophic conditions, iron conditions, light acclimation pigment types and operons, and phosphate adaptation provided.

Strain	Clade	Location	Isolation depth (m)	T_Opt_ (reported)	T_Opt_ (observed)	Thermal ranges (reported)	Cell size (FSCH) range (sampling)	Cell size (FSCH) mean (sampling)	Cell diameter (μm) range	Cell diameter (μm) mean (sampling)	Growth in oligotrophic conditions	Fe conditions	Light acclimation pigment types and operon	Phosphate adaptation
CC9902	IV [42,43]	California Current [38]	5 m	N/A	16°C	N/A	4639–8021	5636	0.64–0.75	0.67	Mesotrophic [42]	**High Fe** [42,44]	3da (*mpeBA*) [45]	High [44]
BL107	IV [39,43]	Balearic Sea [39]	1800 m	24°C [46]	22°C	10.37°C-28.4°C [46]	16126–20826	18999	0.90–0.95	0.93	**Oligotrophic.** [47]	Low Fe adapted [47]	3da (*mpeBA*) [45]	**Potentially low** [44]
CC9311	I [43]	California Current [40]	95 m	22°C [48]	25°C	10°C-25°C [48]	33612–105680	57968	1.50–1.30	1.15	**Mesotrophic** [47,49]	High Fe (coastal) [44,47].	3 (*cpcBA*) [45]	High [44]
ROS8604	I [36,37]	English Channel [41]	0 m	25°C [48] [48,50]	18°C	10°C-28°C [48]	65927–88051	74855	1.20–1.30	1.22	N/A		3a (*mpeBA*) [45]	

#### Particulate Organic Matter (POM) measurements

We measured particulate organic carbon (POC), particulate organic nitrogen (PON), and particulate organic phosphorus (POP), as well as cell enumeration by flow cytometry, following the methods outlined in Garcia et al. (2016) [2]. We sampled after seven doublings or one month to acclimate cells to the temperature conditions. We vacuum filtered POC, PON (150 mL), and POP (50 mL) samples onto pre-combusted GF/F Whatman glass filters (450°C) at 10 psi. We dried particulate organic carbon and particulate organic nitrogen samples at 50–80°C for a minimum of 48 hours and pelletized prior to analysis using a Flash EA 1112 NC Soil Analyzer (Thermo-Scientific, MA). We rinsed filtered particulate organic phosphorus samples with 0.17 M NaSO_4_, immersed the filter in 2 mL of MgSO_4_, dried at 80°C overnight, and combusted at 450°C for 2 hours. We then added 5 mL of 0.2 M HCl and baked the samples at 80–90°C. We measured particulate organic phosphorus samples via colorimetric assay following the Bermuda Atlantic Time-series methodology [51] using a Genesys 10S UV-vis spectrophotometer (Thermo-Scientific) at 885 nm.

### Cell counting

We measured culture cell density every two-three days and immediately prior to sampling using a NovoCyte 1000 flow cytometer (Agilent) (excitation laser 488 nm, emission peak 575 nm) and forward scatter. We assessed growth rate using flow cytometry based on increases in biomass measured across time points using the following equation: *μ* = ln(*CD2*)-ln(*CD1*))/(*T2*-*T1*), in which *CD2* and *CD1* are cell counts in cells/mL^-1^ on the most recent count and the previous count, respectively, and *T2* and *T1* represent time points. We accounted for recent dilutions in accounting for growth rate; we counted cells prior to and after dilutions to ensure growth rate was accurately assessed. We counted the cells at a flow rate of 35 μL/min. To assess heterotrophic populations, we stained the cultures with SYBR Green (Thermo-Fisher) for 15 minutes at room temperature, vortexed them, and counted using the (excitation laser 488 nm, emission peak 520 nm) channel. We recorded duplicate cell counts at each sampling.

### Cell sizing

**Fig 2:**
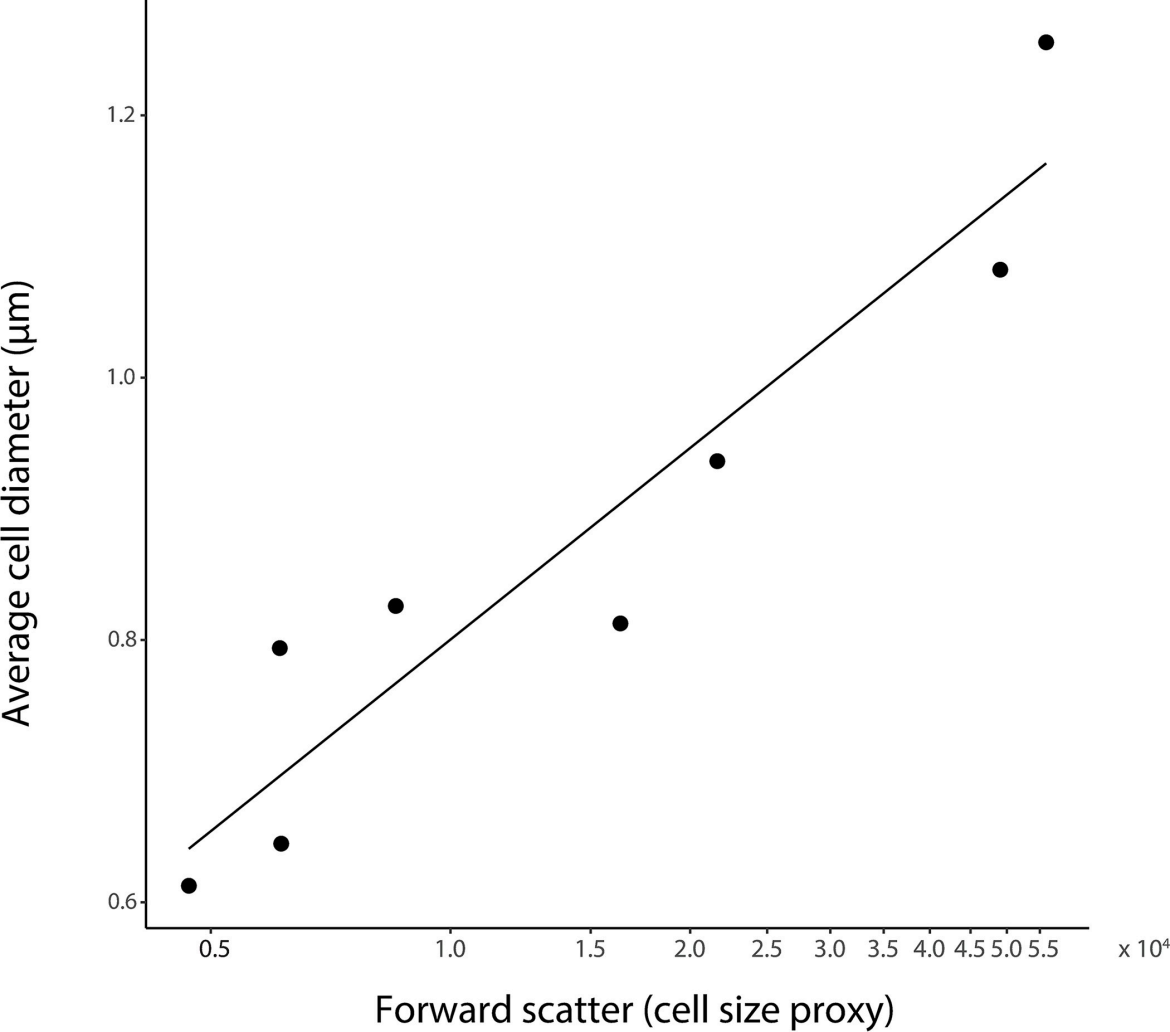
Cell diameter is linked to forward scatter. Cell size measured using flow cytometry (forward scatter, FSCH) and microscopy are highly correlated (r^2^ = 0.83, p = 0.001).

We measured cell diameter by microscopy under oil immersion at 1000x magnification using the Axioplan2 and AxioView 1.4.5 sizing software (Carl Zeiss, Goettingen, Germany) with reference to a staged micrometer (Ted Pella Inc., Redding, CA). To estimate cell diameter, we created a conversion factor determined by plotting the mean observed cell diameters and mean forward scatter values for several strains of *Synechococcus* (Fig 2).

### Additional thermal information

A literature review suggests the optimum growth in clades I and IV are between 22–25°C [46,48]. These temperatures are above the minimum temperature used in our experimental conditions (16°C). Additionally, these conditions allow us to test the upper thermal limits of strains in both Clade I and IV, as well as several temperatures below this upper thermal limit [46,48]. We attempted to probe under which thermal conditions cell growth was enhanced and declined.

### Statistical analyses

We used the R statistical software (www.r-project.org) to perform linear discriminant analyses, analysis of variance, and multivariate analysis of variance analyses. We used the package PHYLIP (https://evolution.genetics.washington.edu/phylip.html) to construct a phylogenetic tree using *rpoc1* sequences [52–57].

## Results

**Fig 3:**
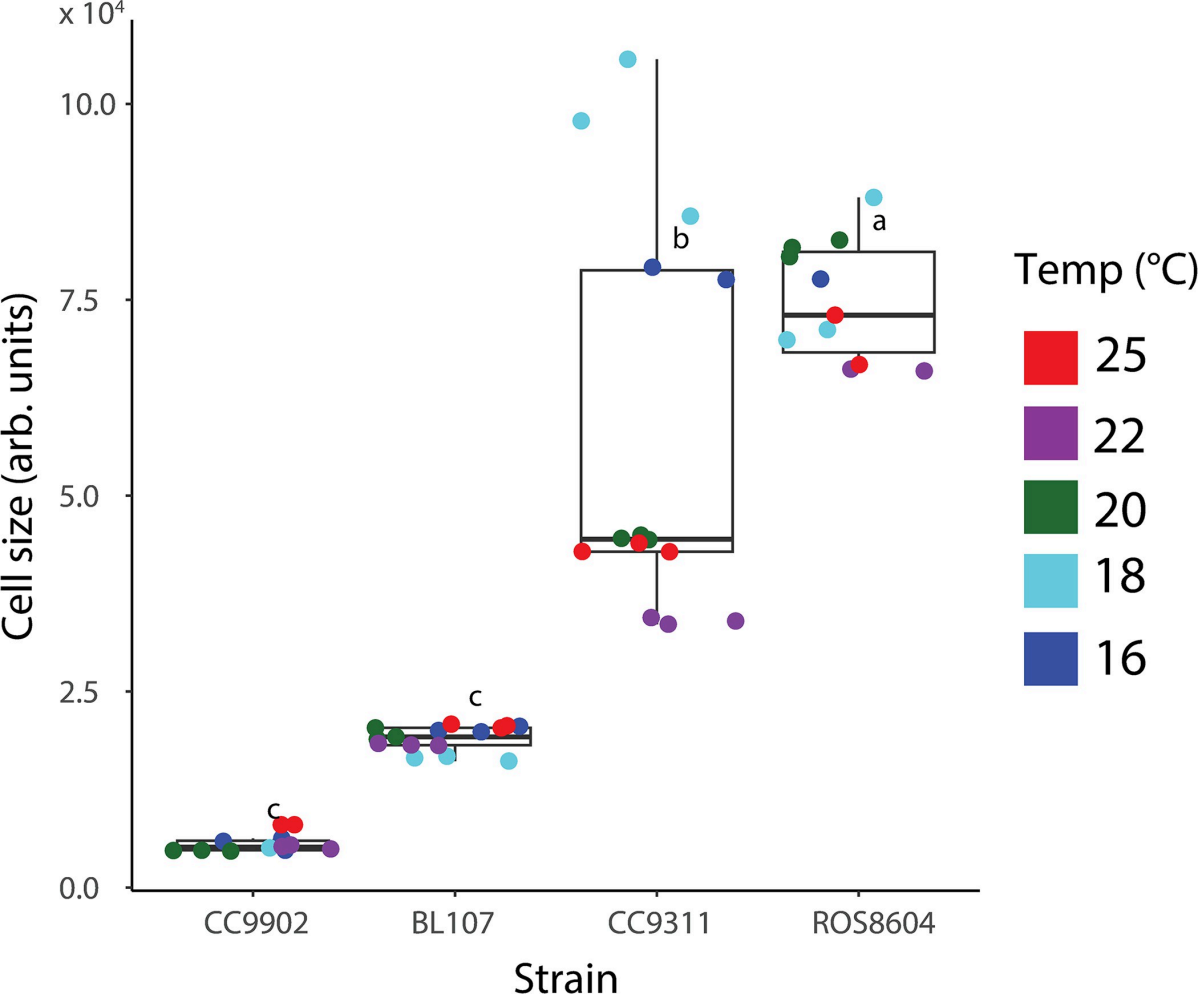
Intraspecific cell size variation among *Synechococcus* strains. Ranges in cell size (FSCH) across strains of *Synechococcus*. We compared the effects of strain on cell size using a one-way ANOVA and Tukey’s honest significance difference (HSD) test, represented by compact letter display (CLD).

We observed distinct ranges in cell size linked to strain identity and phylogeny, whereas temperature had weak effects. We found hierarchical effects on *Synechococcus* cell size. Clade I strains ROS8604 and CC9311 exhibited the largest cell sizes, while clade IV strains BL107 and CC9902 were substantially lower in cell size (Table 1, Fig 3). Inter-clade differences in the effects on cell size were greater than intra-clade effects (Fig 3). However, our results indicated strain served as a secondary phylogenetic effect on cell size; we observed the widest ranges and highest coefficient of variation in cell size in CC9311 (S2 and S7 Tables). In contrast to the effects of clade and strain on cell size, we observed weaker thermal effects on cell size (S1 Table). We found strain exerted a strong effect on cell size and temperature exerted weak effects on cell size.

**Fig 4:**
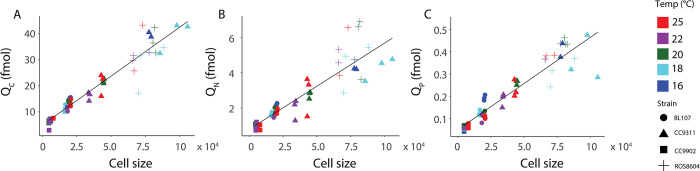
Elemental quotas scale with cell size across *Synechococcus* strains. Fig 4A depicts the carbon cell quota (*Q*_C_) with forward scatter, Fig 4B depicts nitrogen cell quota *Q*_*N*_ with forward scatter, and Fig 4C depicts *Q*_*P*_ with forward scatter. Elemental quotas are depicted in femtomoles (fmol). Point shape represents strain plotted. Point color represents temperature. We performed principal component regressions for each elemental quota and cell size.

We next investigated the relative role of intraspecific trait variation vs. temperature on elemental quotas. The cellular carbon quota (*Q*_*C*_) displayed a strong positive relationship with cell size (Fig 4A). A similar pattern was also seen between cell size and the nitrogen quota (*Q*_*N*_) and the phosphorus quota (*Q*_*P*_) (Fig 4). As cell sizes were unique among the strains, significant relationships between all cell quotas and strain identity were seen (S2 Table). However, we did not find thermal effects alone on *Q*_*P*_, *Q*_*C*_ or *Q*_*N*_, but did observe the presence of thermal effects when other factors, such as strain, were considered for each of these elemental quotas (S4 and S5 Tables). *Synechococcus* represented a diversity of elemental quotas within a limited selection of strains; *Q*_*C*_ ranges from 35.4 fg to 520 fg, *Q*_*N*_ ranges from 10.0 fg to 96.7 fg, and Q_*P*_ ranges from 1.23 fg to 14.7 fg. The *Synechococcus* strains we studied exhibit elemental quota ranges of 14.7 times for *Q*_*C*_, 9.7 for *Q*_*N*_, and 11.4 for *Q*_*P*_ (Fig 4). The degree of variability in elemental quotas is dependent upon lineage; for example, Clade I strains exhibited a wider range in elemental quotas than Clade IV strains (Fig 4A and 4C). We found strain exerted strong effects on elemental quotas, while the role of temperature was weak.

**Fig 5:**
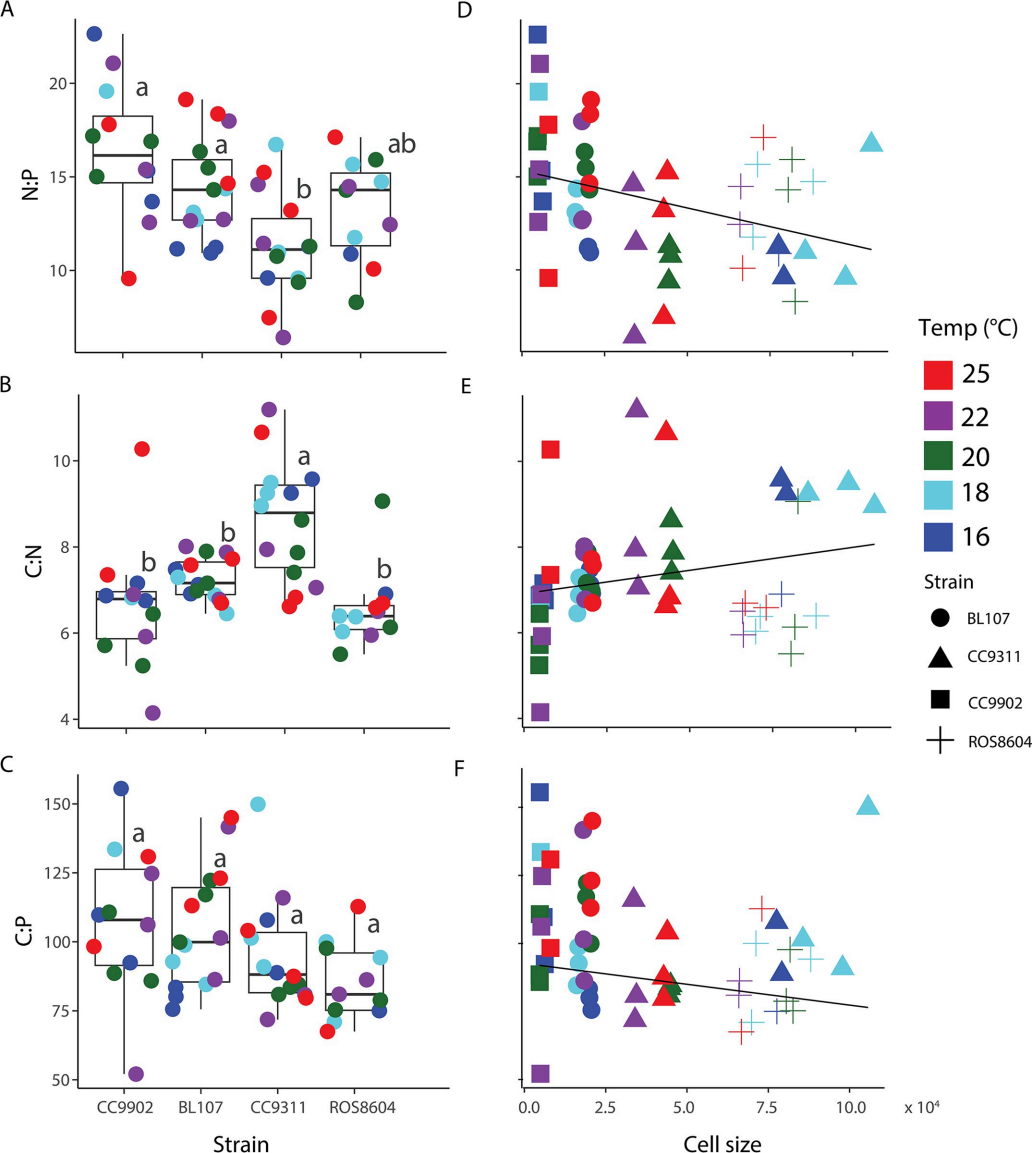
Interactions between *Synechococcus* strain and stoichiometry, and cell size and stoichiometry. We depict effects of strain on N:P (Fig 5A), C:N (Fig 5B), and C:P (Fig 5C) stoichiometry. We depict effects of cell size on N:P (Fig 5D), C:N (Fig 5E), and C:P (Fig 5F) stoichiometry. Color represents temperature (Fig 5A–5C). We compared the effects of strain on stoichiometry (Fig 5A–5C) using a one-way ANOVA and Tukey’s honest significance difference (HSD) test, and are represented by compact letter display (CLD). Elemental ratios were calculated using femtomoles (fmol).

The intraspecific differences in cell size influenced stoichiometry, while the degree and precise nature of the influence varied (Fig 5). We observed significant relationships between strain and both the C:N and N:P ratios (Fig 5B, S2 Table). In contrast, we found no relationship between strain and C:P ratio (Fig 5C, S2 Table). However, when stoichiometric response was examined by clade, we did observe significant differences in N:P and C:P (S3 Table and S3 Fig) but not for C:N ratio (S3 Table and S3 Fig). We found no effects of temperature alone on the N:P, C:N, or C:P (S1 Table). While we did not observe a significant relationship between cell size and C:N or C:P, we did determine a highly significant, inverse relationship between cell size and N:P (Fig 5D–5F, S6 Table). We observed the greatest range in C:N for CC9902 (4.10–10.0), and the lowest range in C:N in BL107 (6.5–8.0). We found CC9902 was the strain with the greatest range in N:P (9.6–22.0), with the smallest range found in BL107 (11–19). We found C:N mean values were 6.30 (CC9902), 7.30 (BL107), 8.60 (CC9311), and 6.60 (ROS8604). We observed the lowest C:P mean (ROS8604, 113.0) for the largest strain examined (CC9902, 155.0; BL107, 145.0; CC9311; 150.0). We found cell size and strain influenced stoichiometry, while thermal effects were weak.

**Fig 6:**
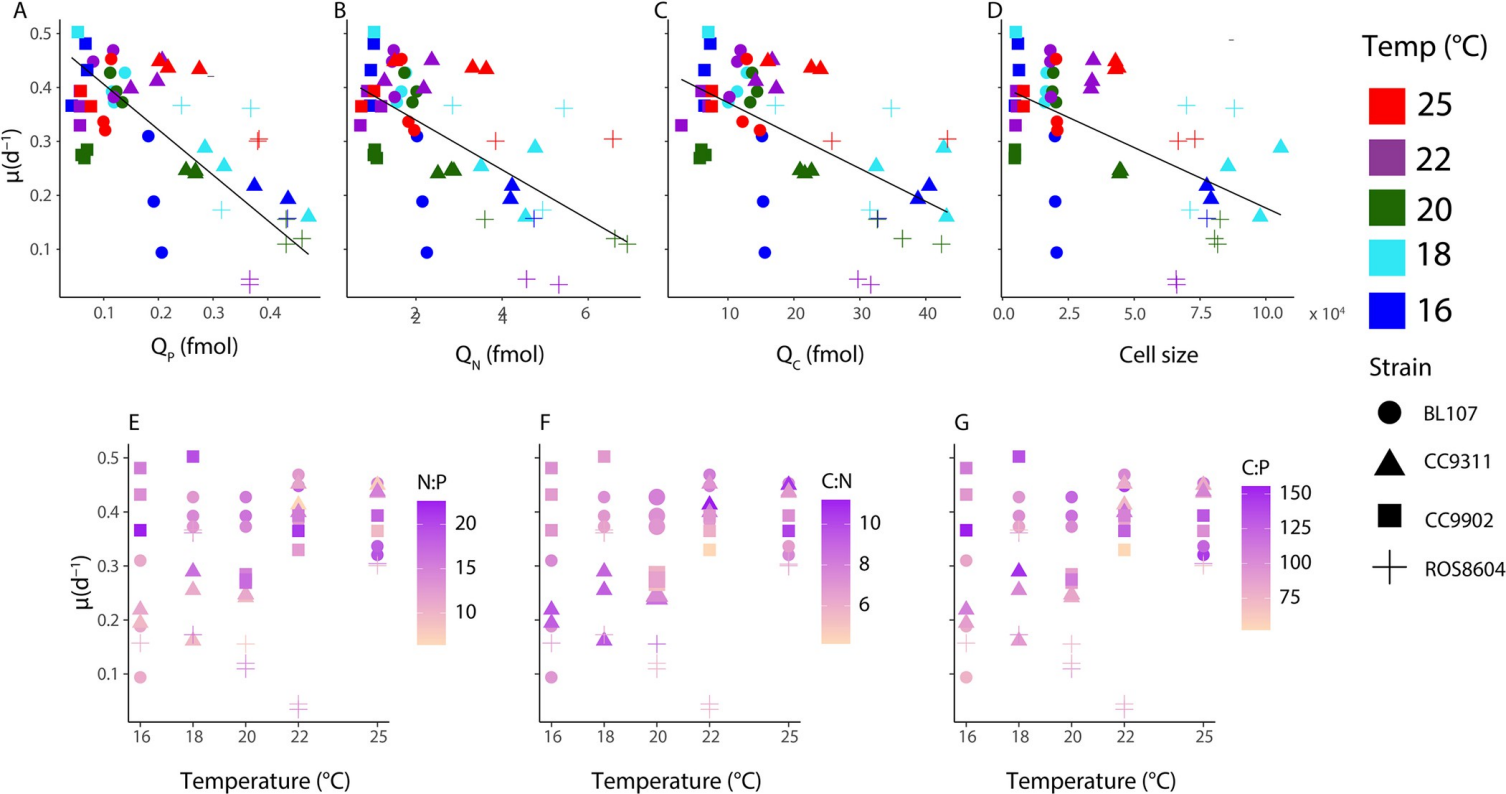
Influence of growth rate on elemental quotas, cell size, and stoichiometry. Growth rate and elemental quotas Q_P_ (Fig 6A), Q_N_ (Fig 6B), and Q_C_ (Fig 6C), cell size (Fig 6D), N:P (Fig 6E), C:N (Fig 6F), and C:P (Fig 6G). Color represents temperature (Fig 6A–6D) or stoichiometry (Fig 5E and 5F). Point shape represents strain. We performed principal component analyses of *μ* and Q_P_ (Fig 6A), Q_N_ (Fig 6B), Q_C_ (Fig 6C) and cell size (Fig 6D), represented by the regression lines. Elemental ratios were calculated using femtomoles (fmol).

We next detected a link between cell size, growth rate and elemental quotas, with a weaker influence of temperature. We assessed the cellular phosphorus quota (Q_P_) and growth rate at different temperatures in Clade IV and I. We observed an inverse relationship between growth rate and Q_P_, Q_N_, and Q_C_ (Fig 6A–6C and S6 Table). Furthermore, growth rate was influenced by temperature only when strain was also considered as a factor likely reflecting the additional effect of adaptation (Fig 6D and S5 Table). Cell size and growth rate varied as a function of both clade and strain, with the two smaller strains (clade IV) demonstrating a greater growth rate than the two larger strains (clade IV) (Fig 6E–6G, S2 and S4 Tables). We found cell size and strain influenced elemental quotas, with a reduced role for temperature.

## Discussion

We observed substantial intraspecific differences in stoichiometric responses among *Synechococcus* strains. We reported higher C:N values relative to previous research conducted using the warm-adapted clade IIIa (WH8012 and WH8103) [33,58]. We found lower C:P ranges in clades I and IV (52–155) relative to those reported for clade IIIa (121–165) [58]. However, while the collective N:P ratios varied between phyla, the grand mean of N:P ratios across all strains of our nutrient-replete *Synechococcus* during exponential growth (13.7) aligned with previously reported values for WH8103 (clade IIIa) (15) and WH7803 (clade V) (13.3) [58,59]. This alignment was only observed during the exponential growth phase (in which we collected samples) emphasizing the importance of growth stage [23,60,61]. Additionally, strains across clade I and IV consistently deviated from the Redfield proportions, supporting the idea that isolates differ from the average ratio of 106:16:1 [5,13]. Our findings suggest phylogeny plays a major role in the stoichiometric response of *Synechococcus*.

Our estimates of cell size clearly partitioned isolates into distinct size classes within the commonly identified range for *Synechococcus* [32]. Our estimates for cell diameter were considerably smaller than some previous estimates [62]. Cell diameter was inversely related to growth rate [12,63], perhaps due to differences in surface area to volume ratios [23]. When combined, low nutrient quotas and high growth rates enable small cells to reach high abundances, particularly in nutrient-poor waters [64]. However, the advantages of being small are counteracted by the costs associated with grazing pressure, which accelerates the trophic transfer of carbon through ecosystems. Conversely, large cells have a higher sinking velocity and relative contribution to carbon export [23]. Modeled estimates of net primary production (NPP), which rely on estimates of *Q*_*C*_ (NPP = *μ* x *Q*_*C*_ x N_cell_) (N_cell_ represents number of cells) and are typically applied to broad phytoplankton groups [6,31]; as differences in cell size may affect differences in carbon flux, our reported variability in cell size within *Synechococcus*—and the associated linkage with *Q*_*C*_*—*highlight the importance of considering strain related variability in *Q*_*C*_. While our results suggest intraclade differences in cell size (S3 Fig and S3 Table) (clade I strains are larger than those of clade IV), we only examined two strains from each clade, and additional research is necessary to more definitively assess this potential relationship. Our results align with previous literature establishing variability in cell size within related phytoplankton groups [65–67]. Our results, which indicate a large range in *Synechococcus* cell size in a small number of strains, suggest it is important to consider this variability in cell size by phylogeny when modeling net primary productivity.

We found temperature exerted little to no direct effect on cell quotas and stoichiometry under nutrient replete serial transfer growth, consistent with previous findings of weak to no thermal effects on growth under nutrient replete conditions [68,69]. Strain-specific temperature responses have been reported in *Prochlorococcus* [60] but the lack of response observed in a previous study on *Synechococcus* was proposed to result from studying *Synechococcus* exclusively at the genus level, and thus only dominant taxa were assessed [70]. As temperature is believed to influence the biodiversity and distribution of phytoplankton, and the optimal C:N:P content is known to vary based on taxonomy or specific oceanic regions [8,20], we also questioned how C:N:P varies across isolates. Although temperature is thought to affect the C:N:P ratios of phytoplankton through the translation-compensation hypothesis [18], we did not find this effect in our assessment of multiple strains of *Synechococcus* across two clades from three distinct regions. We report the observation of indirect thermal effects (e.g., thermal effects as a function of strain). A possible explanation for the lack of strong direct thermal effects may be the lack of our experimental assessment of the lower thermal limits of the four *Synechococcus* strains. Thus, we are unable to make statements about the lower temperature ranges (i.e., approaching thermal limits), where *Synechococcus* may be capable of growth under significant thermal stress. While we did not probe the lower thermal limits of the *Synechococcus* strains used in our experimentation, our results collectively suggest a weak role for thermal effects, which aligns with previous findings [68,69].

We observed some disparities in previously reported and recorded thermal optima (Table 1). Our reported T_Opt_ of CC9311 (22°C) is three degrees lower than that reported by Doré et al. (25°C) [48]. Research conducted by Breton et al indicates BL107 has a T_Opt_ of 24°C [46]. Our reported BL107 T_Opt_ is 22°C; this have arisen as a consequence of our thermal conditions including 22°C and 25°C rather than 24°C. We observed the largest disparity in previously reported and our observed T_Opt_ in ROS8604; previous literature reports T_Opt_ for this strain at 25°C, while our experimentation yielded a T_Opt_ at 18°C [48,50]. Our experimental conditions differed in several ways, which may contribute to the discrepancies in our results [48]. Our cultures were grown on a 12 hour light:dark cycle at 60 μmol quanta m^−2^ s^−1^ in contrast to the continuous lighting conditions previously used [48,50]. Additionally we used modified Artificial Sea Water medium rather than PCR S11 [48,50]. The isolation temperature of ROS8604 was lower than those probed by our experimental conditions (13°C); however, there is reportedly no growth below 14°C [50]. Similarly, the reported thermal minimum for BL107 is 10.37°C [46], and growth declines considerably below 16°C. We grew *Synechococcus* within a broad temperature range of 16–27°C, with 27°C as the upper thermal limit under our experimental conditions. Another explanation is tied to the serial transfer method of culturing *Synechococcus* cells. BL107 and CC9311 demonstrated the greatest strain and temperature differences in growth rate at 16°C (S5 Table). This roughly aligns with findings of Varkey et al. (2016), in which BL107 experiences a decrease in growth rate at lower temperatures, albeit the lowest temperature probed in our experimentation was 16°C rather than 18°C [71]. Here, nutrient levels are elevated possibly resulting in nutrient storage, which may have obscured the role that temperature may play in determining physiological response cell quotas and stoichiometry. Thus, a temperature effect may be more pronounced in polar regions or in conjunction with severe nutrient limitation where nutrient storage is depleted. While we observed some discrepancies in previously reported thermal optima for the *Synechococcus* strains used in our experimentation, our experimental conditions differ in several ways.

The four *Synechococcus* strains differ in their background in terms of adaptation to environmental nutrients, and have distinctive thermal ranges. CC9902 and CC9311 are both mesotrophic strains of *Synechococcus*, adapted to higher concentrations of phosphorus and iron, yet have very distinct physiological responses, which suggests the role of strain is greater than that of adaptation to environmental nutrients in determining environmental responses (Table 1) [42,44,47,49]. This becomes more pronounced as BL107 is an oligotrophic strain, yet its cell size and elemental quotas more closely resemble those of CC9902 (Figs 3–6). As previously reported thermal ranges for these *Synechococcus* strains are similar, and thus we believe it is unlikely we conducted research in drastically different fractions of the upper and lower thermal limits of each strain (Table 1).

The growth rate hypothesis, as articulated in Elser et al. 2000, posits that variations in cellular allocation affecting ribosomal abundance alongside growth in turn affects cellular stoichiometry [72]. The hypothesis states there is an increase in phosphorus and RNA relative to the amounts of nitrogen and proteins that are present [73]. One specific test of the growth rate hypothesis emphasizes the relationship between the maximum growth rate (*μ*_max_) and phosphorus content [74]. Another way of testing the growth rate hypothesis is in terms of physiology, or growth rate (*μ)*; this test examines a given genotype and subjects this clonal organism to variation in environmental conditions; our experimentation took a similar approach, using four *Synechococcus* strains and varying temperature [75]. There remains considerable debate regarding the consistency of the regulation of *μ* across all environmental parameters which may regulate growth, such as nutrients and temperature [3], with some positing the specific variable limiting growth may strongly affect the outcome [3,72]. Research with *Synechococcus* under N and P limitation indicates nucleic acid phosphorus does not drive *Synechococcus* stoichiometry within the ranges of growth examined [2]. Some interpretations of the growth rate hypothesis state that when temperature controls growth, the increased speed of cellular processes may mask the effects on stoichiometry [3]. As we report little to no thermal effects (S1 and S5 Tables), the maximum growth rate should remain the same. Our findings are in agreement with this interpretation of the growth rate hypothesis, and suggest the overall effect of temperature, both within and across strains, has a small physiological effect on growth rate (Fig 6).

Phytoplankton play an essential role in biogeochemical cycling through the ratios of carbon, nitrogen, and phosphorus inside cells. It is crucial to understand the environmental effects on picocyanobacterial traits in order to provide more accurate projections of net primary production (NPP) and their contribution to biogeochemical cycling. We provide evidence that intraspecific variation may play a role in the stoichiometric response and cell size of *Synechococcus*. Our findings of distinct size classes within *Synechococcus* evoke those of other phytoplankton phyla, and thus underscore the importance of considering intraspecific trait variation in biogeochemical and productivity models to generate accurate projections of future changes. Our findings may be used as a foundation for future research for other multi-factorial experiments, such as determining the role of temperature and nutrient supply on these strains and clades of phytoplankton in response to projected change.

## Supporting information

S1 Fig Thermal effects on elemental quotas and stoichiometry of *Synechococcus*. Statistical analysis was performed using ANOVA. Points are colored by strain. Horizontal lines represent the median value for elemental quotas or stoichiometry. Regions highlighted in yellow represent the experimental conditions at which the highest growth rate was recorded.
(TIF)

S2 Fig Continued biomass growth past sampling date without addition of further nutrient supply indicates *Synechococcus* cultures used are nutrient replete. Strain is represented by point color, sampling day (BL107, CC9311: day 14, ROS8604, CC9902: day 40) is highlighted in yellow.
(TIF)

S3 Fig Cell size is linked to *Synechococcus* clade. Ranges in cell size (FSCH) across clade IV and I of *Synechococcus*. We compared the effects of clade on cell size using a one-way ANOVA and Tukey’s honest significance difference (HSD) test and are represented by compact letter display (CLD).
(TIF)

S4 Fig N:P and C:P is linked to *Synechococcus* clade. Ranges in stoichiometry across clade IV and I of *Synechococcus*. We compared the effects of clade on stoichiometry using a one-way ANOVA and Tukey’s honest significance difference test (HSD), and are represented by compact letter display (CLD).
(TIF)

S1 Table One-way ANOVA (temperature) analyses for *Synechococcus* experiments. Significance is denoted with an asterisk (*).
(CSV)

S2 Table One-way ANOVA (strain) analyses for *Synechococcus* experiments. Significance is denoted with an asterisk (*).
(CSV)

S3 Table One-way ANOVA (clade) analyses for *Synechococcus* experiments. Significance is denoted with an asterisk (*).
(CSV)

S4 Table Two-way ANOVAs for *Synechococcus* experiments (clade, temperature). We performed two-way ANOVAs to assess the effects of multiple independent variables on a dependent variable. Significance is denoted with an asterisk (*).
(CSV)

S5 Table Two-way ANOVAs for *Synechococcus* experiments (strain, temperature). We performed two-way ANOVAs to assess the effects of multiple independent variables on a dependent variable. Significance is denoted with an asterisk (*).
(CSV)

S6 Table Principal component regressions and logarithmic regression for *Synechococcus* experiments. Significance is denoted with an asterisk (*).
(CSV)

S7 Table Coefficient of variation in cell size of *Synechococcus* strains. (CSV)

S8 Table (CSV)

S1 File (ZIP)
