# Supplementary figures and images for: Intraspecific trait variation modulates the temperature effect on elemental quotas and stoichiometry in marine Synechococcus
Source: PLoS One. 2024 Mar 18;19(3):e0292337. doi: 10.1371/journal.pone.0292337 (PMC10947687; doi:10.1371/journal.pone.0292337)

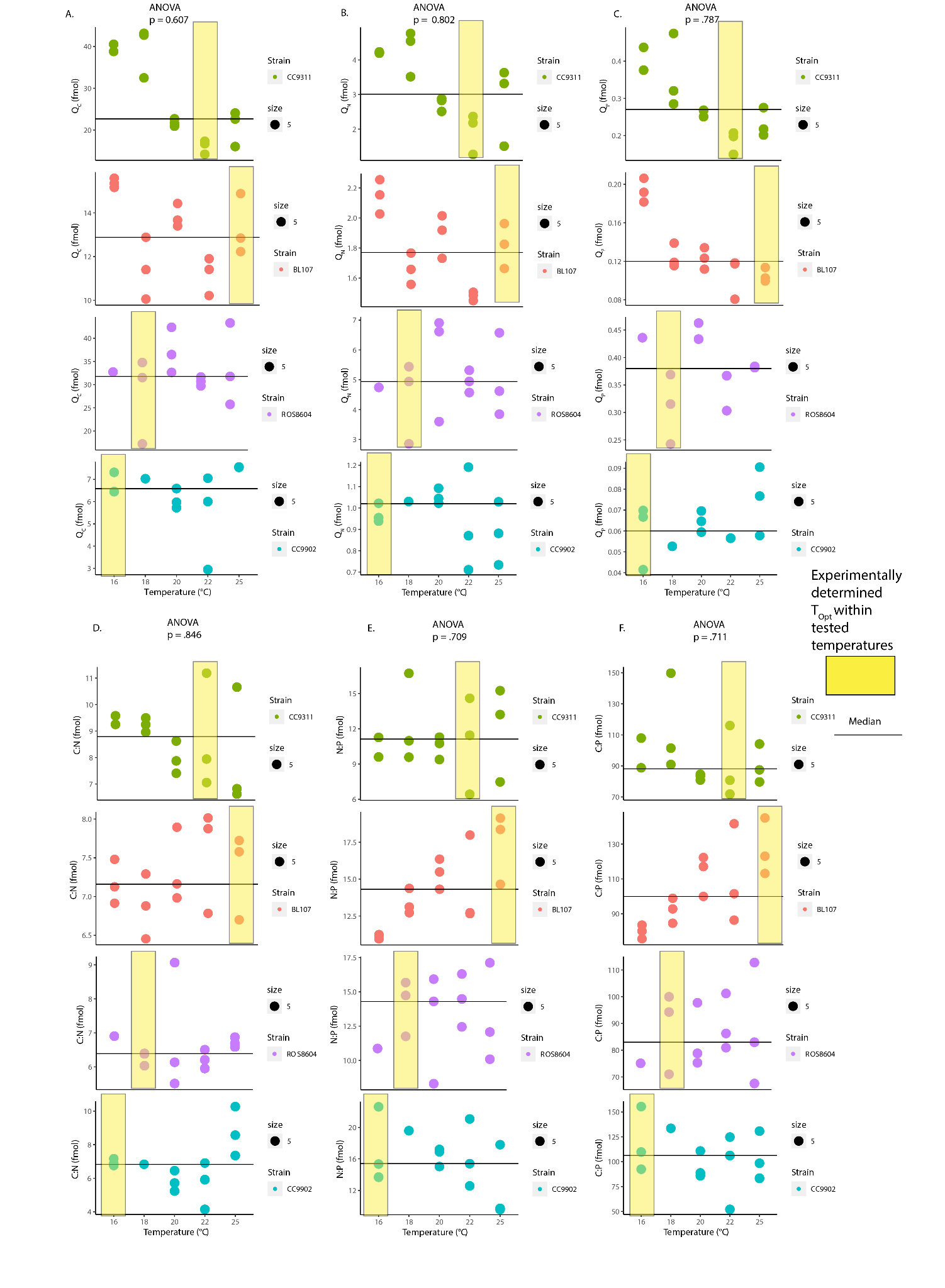

Supplement: S1 Fig — Statistical analysis was performed using ANOVA. Points are colored by strain. Horizontal lines represent the median value for elemental quotas or stoichiometry. Regions highlighted in yellow represent the experimental conditions at which the highest growth rate was recorded. (TIF) [file pone.0292337.s001.tif]

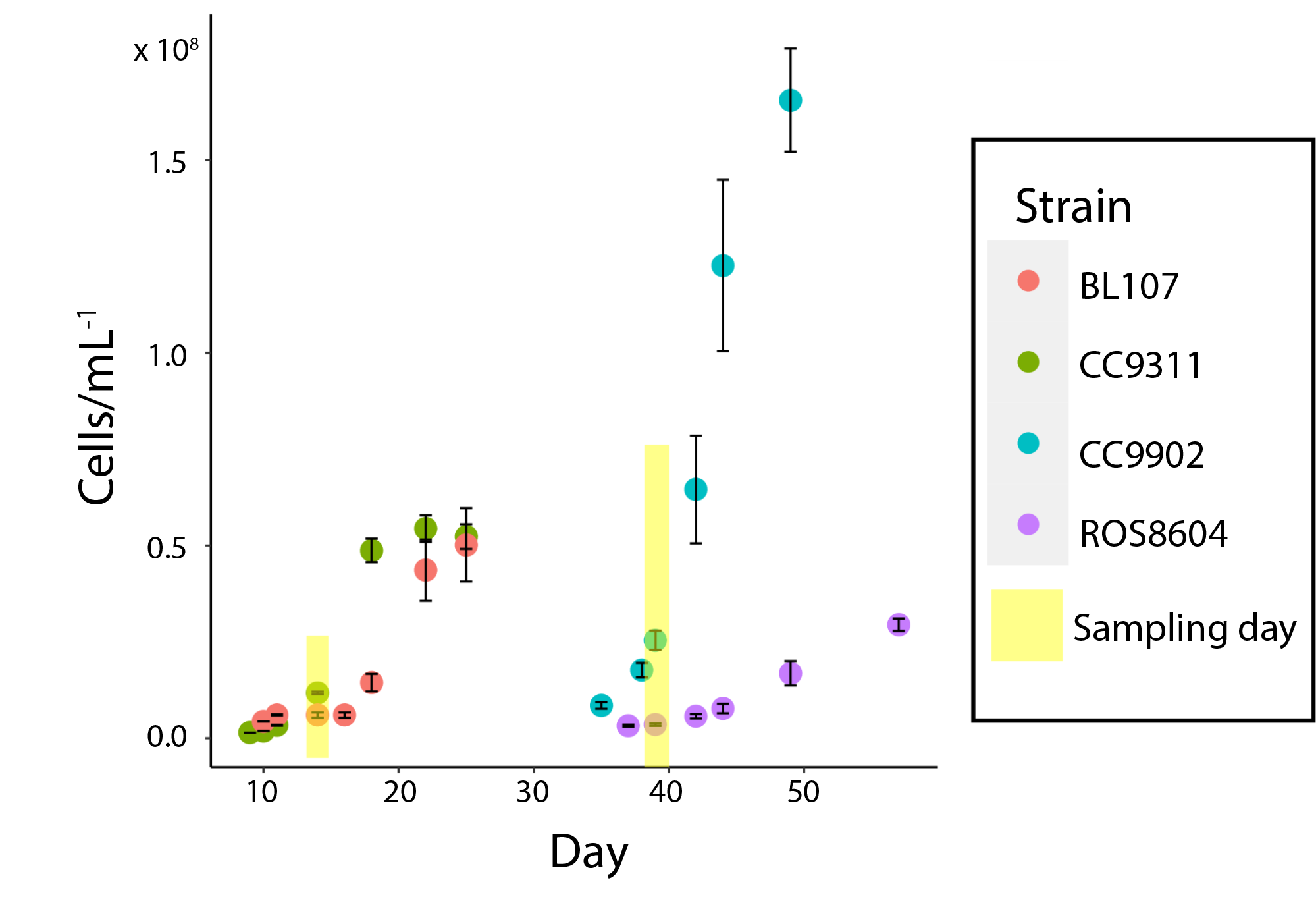

Supplement: S2 Fig — Strain is represented by point color, sampling day (BL107, CC9311: day 14, ROS8604, CC9902: day 40) is highlighted in yellow. (TIF) [file pone.0292337.s002.tif]

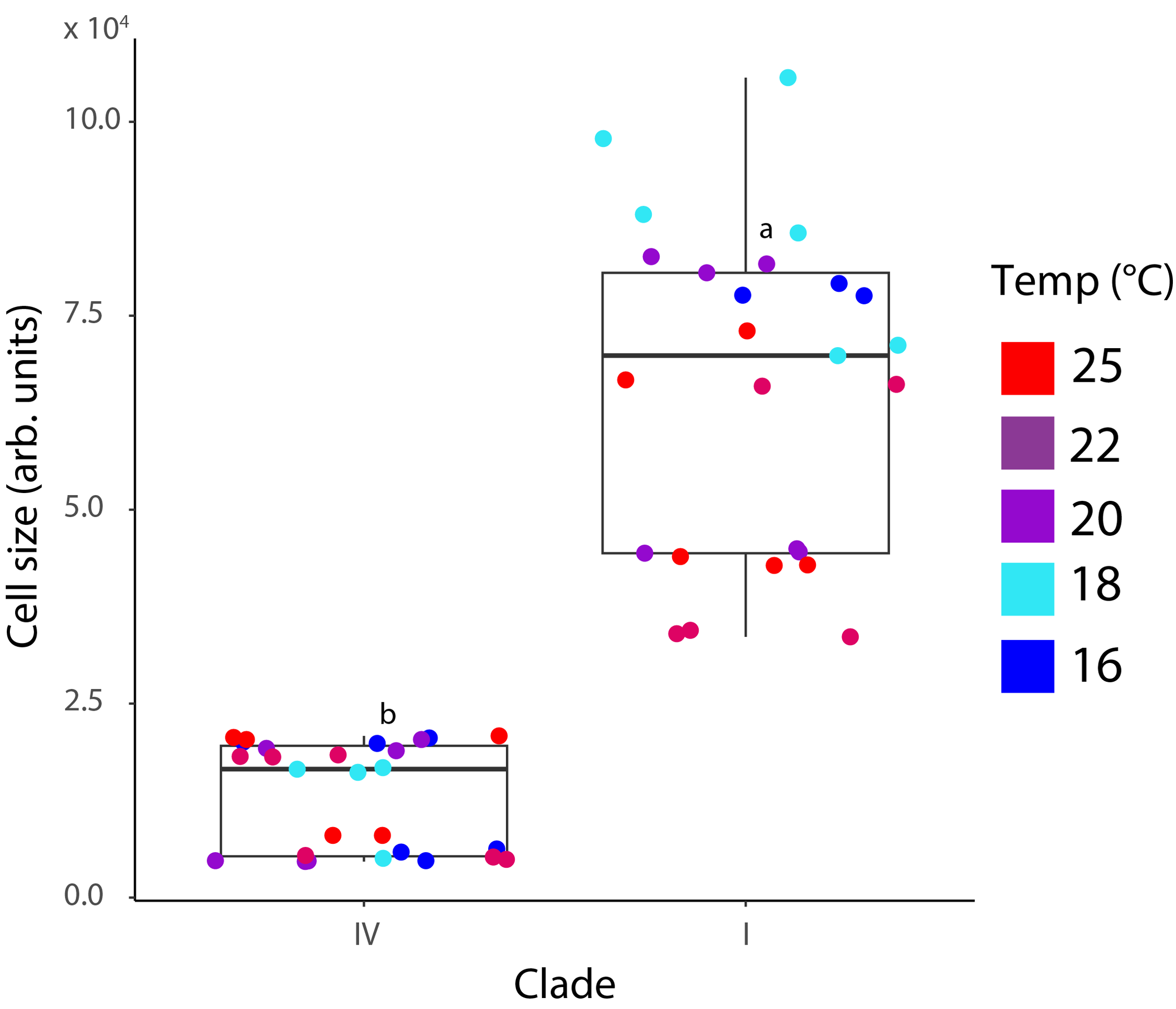

Supplement: S3 Fig — Ranges in cell size (FSCH) across clade IV and I of Synechococcus. We compared the effects of clade on cell size using a one-way ANOVA and Tukey’s honest significance difference (HSD) test and are represented by compact letter display (CLD). (TIF) [file pone.0292337.s003.tif]

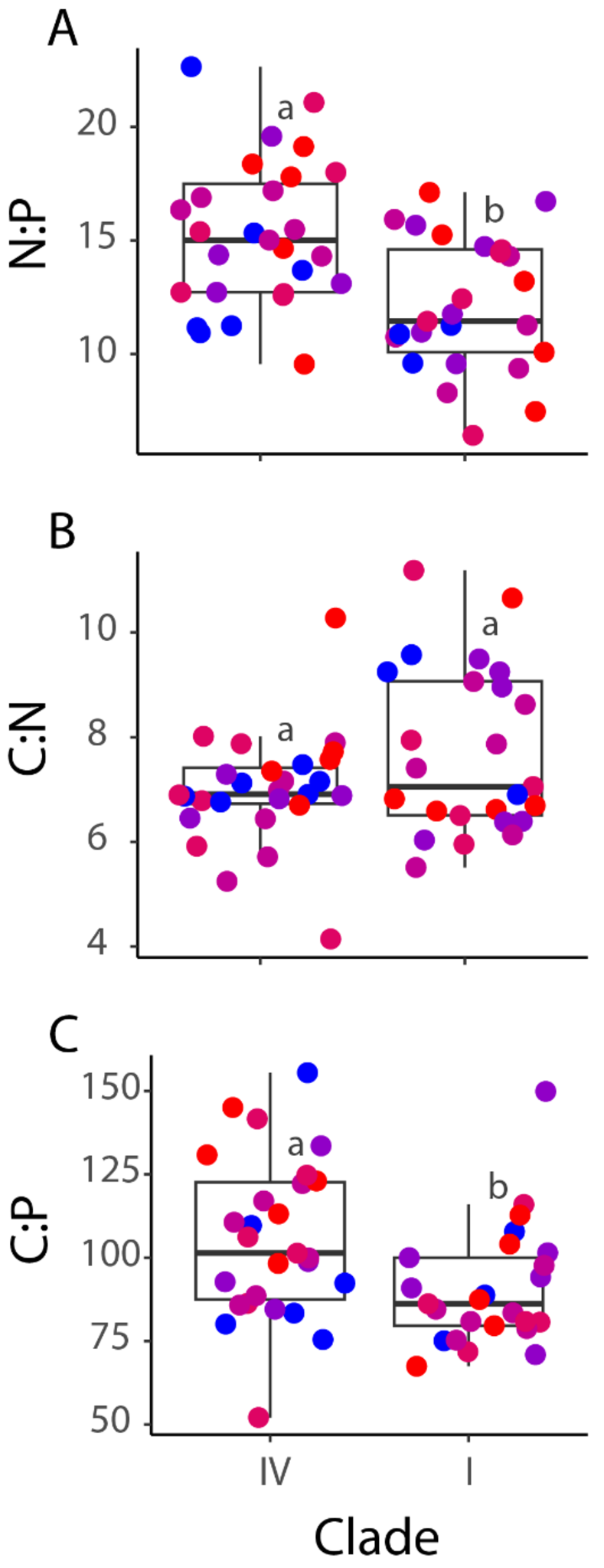

Supplement: S4 Fig — Ranges in stoichiometry across clade IV and I of Synechococcus. We compared the effects of clade on stoichiometry using a one-way ANOVA and Tukey’s honest significance difference test (HSD), and are represented by compact letter display (CLD). (TIF) [file pone.0292337.s004.tif]
